# Conjugated polymer nanoparticles for effective siRNA delivery to tobacco BY-2 protoplasts

**DOI:** 10.1186/1471-2229-10-291

**Published:** 2010-12-30

**Authors:** Asitha T Silva, Alien Nguyen, Changming Ye, Jeanmarie Verchot, Joong Ho Moon

**Affiliations:** 1 Department of Entomology and Plant Pathology, Oklahoma State University, Stillwater, OK, USA 74078; 2 Department of Chemistry and Biochemistry, Florida International University, Miami, FL 33199, USA

## Abstract

**Background:**

Post transcriptional gene silencing (PTGS) is a mechanism harnessed by plant biologists to knock down gene expression. siRNAs contribute to PTGS that are synthesized from mRNAs or viral RNAs and function to guide cellular endoribonucleases to target mRNAs for degradation. Plant biologists have employed electroporation to deliver artificial siRNAs to plant protoplasts to study gene expression mechanisms at the single cell level. One drawback of electroporation is the extensive loss of viable protoplasts that occurs as a result of the transfection technology.

**Results:**

We employed fluorescent conjugated polymer nanoparticles (CPNs) to deliver siRNAs and knockdown a target gene in plant protoplasts. CPNs are non toxic to protoplasts, having little impact on viability over a 72 h period. Microscopy and flow cytometry reveal that CPNs can penetrate protoplasts within 2 h of delivery. Cellular uptake of CPNs/siRNA complexes were easily monitored using epifluorescence microscopy. We also demonstrate that CPNs can deliver siRNAs targeting specific genes in the cellulose biosynthesis pathway (*NtCesA-1a *and *NtCesA-1b)*.

**Conclusions:**

While prior work showed that *NtCesA-1 *is a factor involved in cell wall synthesis in whole plants, we demonstrate that the same gene plays an essential role in cell wall regeneration in isolated protoplasts. Cell wall biosynthesis is central to cell elongation, plant growth and development. The experiments presented here shows that *NtCesA *is also a factor in cell viability. We show that CPNs are valuable vehicles for delivering siRNAs to plant protoplasts to study vital cellular pathways at the single cell level.

## Background

Post transcriptional gene silencing (PTGS) is a cellular mechanism that regulates gene expression in the cytoplasm [1,2]. In this mechanism, mRNA is reverse transcribed to produce long double-stranded RNA which is then digested by the Dicer enzyme to produce smaller fragments of discrete sizes. There are two classes of silencing RNAs, known as microRNA (miRNA) and small interfering RNA (siRNA) [2-4]. miRNAs are endogenous noncoding small RNAs that are 18 to 25 nucleotide (nt) long and function to repress mRNA translation or target mRNA for degradation [5]. miRNAs contribute to the regulation of gene expression for development, responses to external stressors, and cell cycle control [6]. siRNAs are 21 to 24 nt long and derive from mRNAs or viral RNAs [7]. Endoribonuclease-containing complexes, known as RNA-induced silencing complexes (RISCs), incorporate the miRNAs and siRNAs which act to guide the RISCs to homologous cellular mRNAs, targeting them for degradation [8-10]. PTGS acts to prevent translation of targeted gene products and effectively knock out gene expression.

PTGS has been harnessed by plant biologists as a tool to knock down expression of essential genes during investigations of their role in metabolism in whole plants and protoplasts [11]. Viral vectors are commonly used for delivery of siRNAs or miRNAs into plants. Viral vectors offer the advantage of transiently and directly expressing the siRNA without relying on plant transformation. The most widely used vector for delivery of siRNAs is the bipartite *Tobacco rattle virus *(TRV) [12,13]. The *Cabbage leaf curl virus *(CbLCV) vector was recently developed for expressing synthetic and endogenous miRNAs in plants [15]. For TRV and CbLCV vectors, the genomic cDNA was introduced into T-DNA vectors and used for *Agrobacterium tumefaciens *delivery by infiltration into leaves [12,14,15]. Entire or partial gene sequences are expressed from the TRV vector while artificial miRNA precursors have been expressed from the CbLCV genome which share homology to the host gene targeted for silencing. As the virus spreads systemically, virus-derived siRNAs or miRNAs guide the RISC complex to degrade target transcripts. Because most viruses are limited in their host range, additional viral vectors are being developed for small RNA delivery to diverse plant species. Another drawback of viral vectors is that they do not uniformly infect all tissues, although they might spread systemically. In addition, the phenotype attributed to PTGS is mixed with the onset of virus symptoms which include mosaic pattern of disease and mild leaf curling.

Protoplasts are isolated from plant suspension cells or intact tissues by treating them with cell wall degrading enzymes. Protoplasts have intact plasma membranes but are fragile because of the loss of the cell wall. Protoplasts are typically employed in cell culture assays for physiological, biochemical, and molecular studies of plant cell functions. They can survive for up to 72 h in culture with some loss of viability, but in suitable media, cultured protoplasts can regenerate cell walls, undergo cell division, and even regenerate plants [16-18]. Gene transfer or siRNA delivery into protoplasts is typically achieved using electroporation which involves applying an electric field to protoplasts held in a cuvette. Electroporation increases the plasma membrane permeability and enables nucleic acid penetration [19,20]. One important drawback is the significant loss of viable protoplasts during electroporation. Depending on the source of protoplasts (i.e. plant species as well as the source tissues such as leaf, cotyledons, young shoots, suspension cells) and the voltage applied, losses of 50% viable protoplasts can occur [19,21].

CPNs are intrinsically fluorescent nanoparticles fabricated by ultrafiltration of amine-containing conjugated polymers (CPs) treated with an organic acid in aqueous phases [22,23]. These organic nanoparticles are stably suspended in water (without evidence of precipitation) for several months under ambient storage condition. The spectral properties of CPN were previous described and the absorption maximum is centered at 438 nm and emission maximum is at 483 nm [23]. Dynamic light scattering measurements revealed the hydrodynamic radius of CPNs is around 60-80 nm, depending on molecular weights and organic acid treatments. CPNs are positively charged, and exhibit affinity with negatively charged biological substances such as nucleic acids [22]. CPNs have the potential to act as a protective, efficient and self-tracking transfection agent for RNA interference experiments. The complexation of siRNA with CPNs can improve RNA stability by protecting them from RNAse degradation, as reported for other polymeric siRNA delivery systems [24]. In addition, various mammalian cells take up CPNs without toxic effects. Given their ability to traverse cellular membranes we postulated that CPNs can be used to visually monitor siRNA internalization using a simple complexation between CPNs and siRNAs.

In this study, we examine the delivery of siRNA to protoplasts using CPNs. We employed siGLO Red siRNA, which is a commercially available, red fluorescent dye-labeled siRNA. We found that CPN is a potent transfection agent that can be used to deliver and visually monitor the uptake of abundant siRNAs to plant protoplasts. We also demonstrate that CPNs can deliver siRNAs targeting specific genes in the cellulose biosynthesis pathway (*NtCesA-1a *and *NtCesA-1b*). Cellulose synthase is a multigene family that is not fully characterized in tobacco. *NtCesA-1a *and *NtCesA-1b *are related accessions but *NtCesA-2 *is a distinct gene with 80% homology to *NtCesA-1a*. In a prior report, a PVX vector containing *CesA-1a *gene fragments were delivered to intact plants. The outcomes of *CesA-1a *silencing included reduced cellulose content of the plant cell walls, but this was also accompanied by an increase in homogalacturonan and decreased esterification of pectic polysaccharides in silenced plants [25]. Here, we show that CPNs deliver *NtCesA-1 *siRNAs that effectively knockdown cell wall biosynthesis during the early stages of synthesis in protoplasts indicating that *NtCesA-1 *is crucial. Therefore, CPNs provide an attractive alternative for siRNA delivery and gene knockout in cultured protoplasts.

## Results

### CPNs are taken up by BY-2 protoplast but not by intact cells

**Figure 1:**
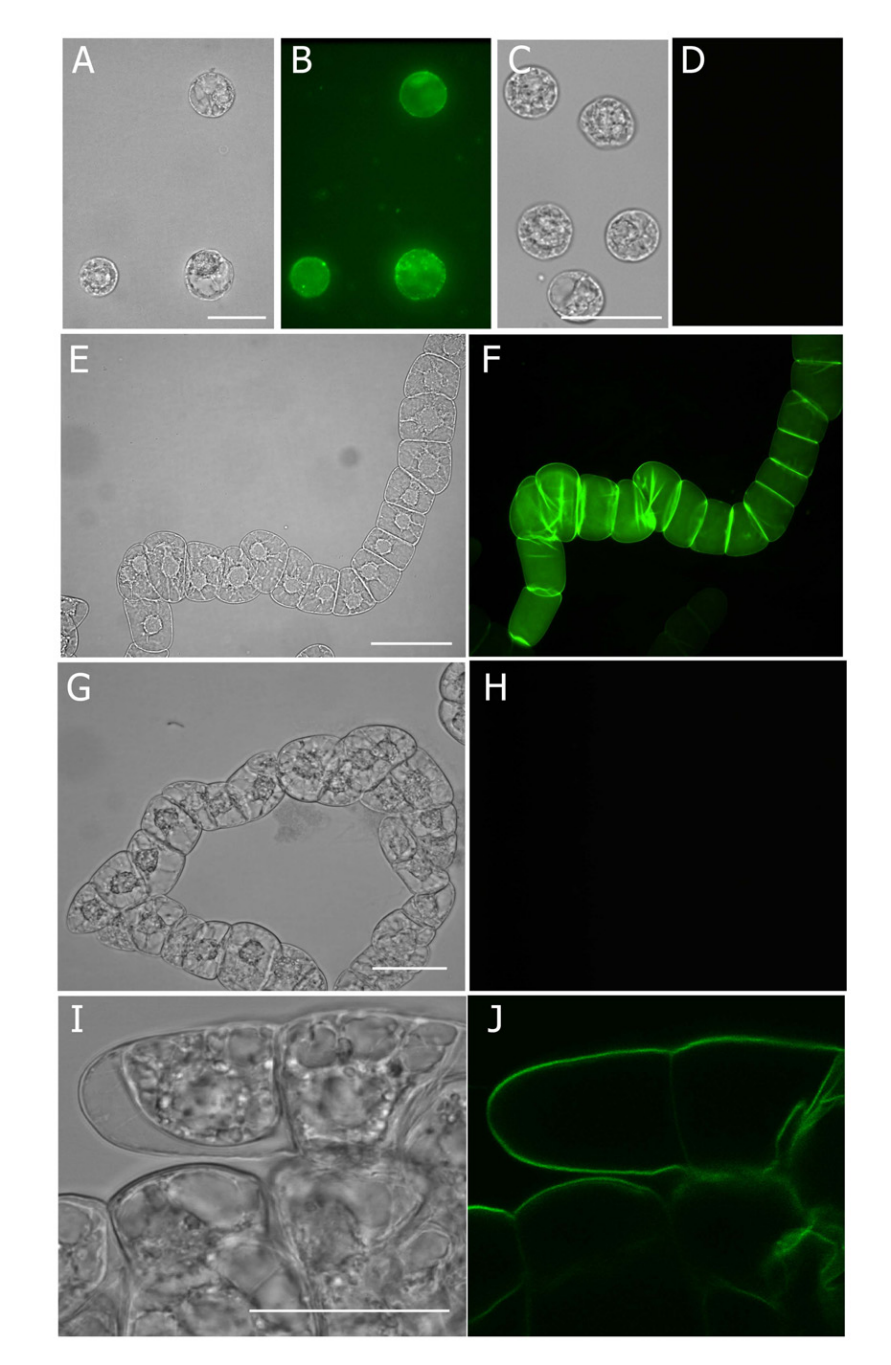
**CPN-treated BY-2 cells and protoplasts**. Panels show bright field and fluorescence images. **(A,B) **BY-2 protoplasts were incubated for 2 h with 10 μM CPNs or **(C, D) **left untreated. **(E, F) **Chains of attached BY-2 cells treated with 10 μM CPNs for 2 h. Green fluorescence is greatest at the cross walls suggesting that CPNs attach to the cell walls and do not penetrate the interior. **(G, H) **Images of untreated BY-2 cells at 2 h. **(I, J) **Confocal images of intact BY-2 cells treated with 10 μM CPNs at 24 h. Experiments were repeated with similar results. Single optical section through the center of the cell shows fluorescence along the cell wall and does not penetrate the interior. Scale bars equal 50 μm.

BY-2 protoplasts were incubated with various concentrations of CPNs (5, 10, 15, and 25 μM) for either 2 or 24 h followed by counting cells to determine the proportion of green fluorescing cells under the microscope (n = 400). At 2 h following the delivery of 5 μM CPNs to the culture medium, 35% of protoplasts showed green fluorescence, while 60-75% of protoplasts treated with 10-25 μM CPNs showed fluorescence. At 24 h, the proportion of green fluorescent protoplasts increased to 50% following treatment with 5 μM CPNs and 79-90% following treatment with 10-25 μM CPNs (Figure 1A, B). Importantly, untreated samples did not fluoresce green (Figure 1C, D). Optical sections obtained by laser-powered confocal microscopy confirming internal localization of CPNs (data not shown). Fluorescence was mainly cytosolic, and did not appear to be nuclear (Figure 1A, B).

We treated intact BY-2 suspension cells with 5, 10, 15, and 20 μM CPNs (Figure 1E, F) and the plant cell wall was a barrier to uptake. Optical sections obtained by laser-powered confocal microscopy of CPN-treated BY-2 cells showed the fluorescence remained bound to the cell surface even after 24 h of incubation (Figure 1I, J). Untreated samples showed no green fluorescence (Figure 1G, H).

### Uptake of CPNs is reminiscent of endocytic pathway

**Figure 2:**
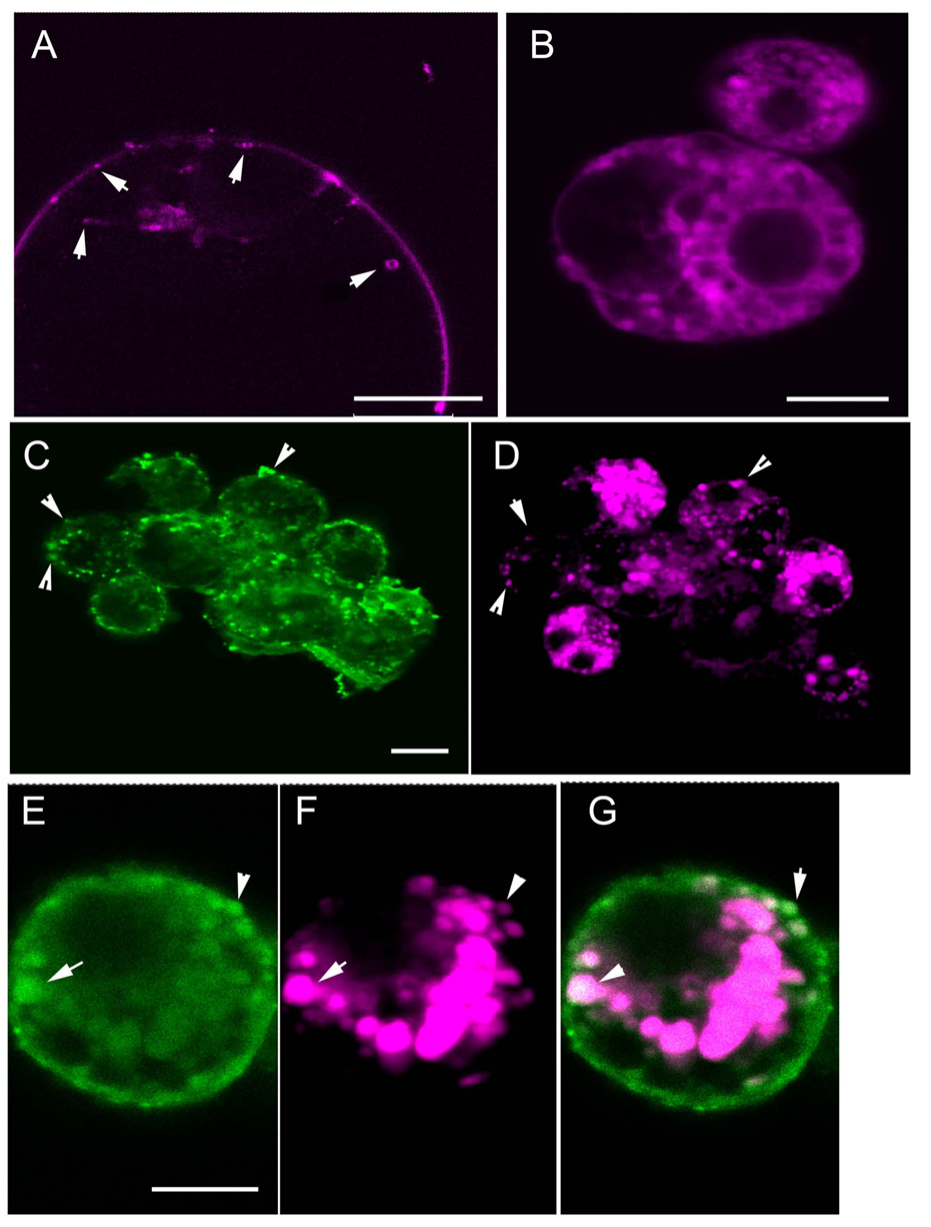
**CPN and FM4-64 treated BY-2 protoplasts examined using confocal microscopy**. **(A) **Protoplast that was treated with medium (no CPNs) and then FM4-64 for 10 min or **(B) **more than 20 min. FM4-64 fluorescence is in the plasma membrane and vesicles budding from the plasma membrane at 10 min. Following a 20 min or longer incubation, red fluorescence is in the plasma membrane, perinuclear membranes, and intracellular vesicles. Arrow heads point to vesicles budding at the plasma membrane and in the cortical region. **(C, D) **Green and red fluorescent images of protoplasts treated with CPNs and then FM4-64 for 10 min. Arrow heads point to vesicles along the plasma membrane that contain both green and red fluorescence. There are a greater number of red than green fluorescent vesicles. However, most green granules also contain red fluorescence. **(E, F, G) **Protoplasts were treated with CPNs and then FM4-64 for 20 min showed green and red fluorescence in vesicles along the periphery of the cell. Repeated experiments showed similar outcomes. Arrows point to examples where green and red fluorescence overlap. Scale bars equal 20 μm.

In a recent study, positively charged nanogold particles were transferred at the plasma membrane to the early endosome and then into larger peripheral vesicles [26]. The role of the large peripheral vesicles and the destination beyond these vesicles in plant cells has not been described, although there is some speculation that these are prevacuolar vesicles [26]. In protoplasts, the CPNs often occur in cytoplasmic granules and we hypothesized that these are either aggregates of nanoparticles, endocytic vesicles, or both. Given that CPNs have positive charge, they might enter the endocytic pathway, similar to the charged nanogold particles, and then be released into either the cytoplasm or another membrane bound compartment. Therefore we employed FM4-64, which is a membrane-staining dye for live cell imaging, to track endocytic vesicles budding from the plasma membrane in CPN-treated protoplasts [27]. Untreated protoplasts stained with FM4-64 for 10 min, showed uniform red fluorescence in the plasma membrane, and bright spots where vesicles begin to form (Figure 2A, arrowheads). Few internal vesicles appear. Following staining for 20 min, the red fluorescence occurred in prevacuolar and vacuolar membranes (Figure 2B).

Protoplasts were incubated with 10 μM of CPNs for 24 h followed by incubation with FM4-64 for 10 -30 min. Green and red fluorescence co-localized in vesicles at the cell margin and internally. There was a profusion of red fluorescent vesicles, which was not seen in control samples (not treated with CPNs). The exogenous application of CPNs stimulated either the production of endosomes by the cell or dye uptake by an alternative route (compare Figure 2A, B, and 2D). We followed the transition of FM4-64 dye over time. After 10 min of staining, green and red fluorescence appeared in granules along the plasma membrane (Figure 2C, D, arrowheads). Green and red fluorescence then co localized in large peripheral vesicles around 30 min later (Figure 2E-G). The larger peripheral bodies (Figure 2E-G) resembled prevacuolar vesicles (such as multi-vesiculate bodies). Given that proteins taken up by the early endosome can be transported either to the Golgi apparatus or prevacuolar vesicles, the pattern of FM4-64 staining is expected. Figure 2 shows a pattern of CPNs transitioning from small granules at the cell surface to larger vesicles, argues that CPNs follow the same uptake pathway as FM4-64. While further high resolution experiments are needed to define the various internal compartments, the pattern of CPN-uptake suggests a membrane mediated route rather than diffusion across the plasma membrane.

### 5-25 μM CPNs are nontoxic to BY-2 protoplasts

**Figure 3:**
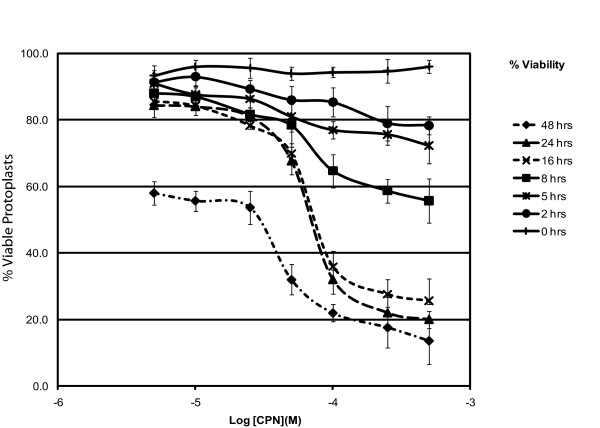
**Protoplast viability was determined following specific incubation with various concentrations of CPNs**. Protoplasts were cultured for various times between 0 and 48 h following treatment with the following CPN concentrations: 5, 10, 25, 50, 100, 250 and 500 μM. The percentage of viable protoplasts was determined using propidium iodide staining at 0, 2,5,8,16,24 and 48 h. The data were expressed as the average % viability at each time point for log molar concentration of CPNs taken from three independent experiments.

Reports indicate that cadmium-based nanoparticles have the potential to be cytotoxic to mammalian cells. The cytotoxic potential can be influenced by the particle sizes and concentrations, distribution to different regions of the cell, or liberation of Cd^2+ ^from the nanoparticle lattice [28,29]. Although CPNs are polymers that do not contain Cd^2+ ^and are distributed in the cytoplasm, we cannot rule out the possibility of a concentration dependent cytotoxicity. Therefore, propidium iodide staining was employed to measure the cytotoxic impact of various concentrations (0, 5, 10, 25, 50, 100, 250, and 500 μM) of CPNs following treatments for 0, 2, 5, 8, 16, 24, and 48 h (Figure 3). The average percent of viable protoplasts at each time point was calculated for three replicate experiments.

Concentrations of 5-25 μM are non-toxic to protoplast and do not significantly reduce their viability. Typically, following preparation of BY-2 protoplasts from intact cells, we noted 90-96% viable protoplasts during the first 8 h of culture. This declines to 85% at 24 h and then 60% at 48 h (Table 1). 80-95% of BY-2 protoplasts were viable during the first 8 h of culture following treatment with 5, 10, and 25 μM of CPNs (log molar concentrations of -4.3 to -5.3), which is comparable to untreated protoplasts. Protoplast viability following CPN treatment further declined to 81-86% at 24 h and 53-60% at 48 h. Thus, the average percent viability is similar over time among the CPN-treated (5-25 μM) and untreated protoplasts.

**Table 1:** Effect of various concentrations of CPNs on viability of BY-2 protoplasts at 0 and 24 h

Conc (μM)	0 h	24 h
**0**	95.7 ± 2.5	85.7 ± 3.2
**5**	93.3 ± 3.0	84.3 ± 3.5
**10**	96.0 ± 2.0	84.0 ± 2.7
**25**	95.7 ± 3.0	81.0 ± 2.7
**50**	94.0 ± 2.0	67.7 ± 4.2
**100**	94.3 ± 1.5	32.0 ± 4.4
**250**	94.7 ± 3.5	22.0 ± 5.2
**500**	96.0 ± 2.0	20.0 ± 2.7

Low concentrations of (5-25 μM) CPNs have minimal impact on protoplast viability at 24 h.

Concentrations ≥ 50 μM CPNs cause significant loss of BY-2 viability at 24 h.

Concentrations over 50 μM (log molar concentrations of -3.3 to 4.0) cause the proportion of viable protoplasts to decline profoundly after 8 h (Figure 3; Table 1). The average percentage of intact protoplasts treated with 50 μM CPNs was 68% at 24 h and 32% at 48 h. For concentrations of 100- 500 μM the average percent viability was 20 to 32% at 24 h and 14 to 22% at 48 h (Figure 3; Table 1). These data show that the concentrations of CPNs (5-25 μM) used in the prior experiments for visualizing uptake are essentially non-toxic to BY-2 protoplasts but excessive amounts of polymer can be detrimental.

### CPNs deliver both siGLO Red and *NtCes1-A *siRNAs to protoplasts

**Figure 4:**
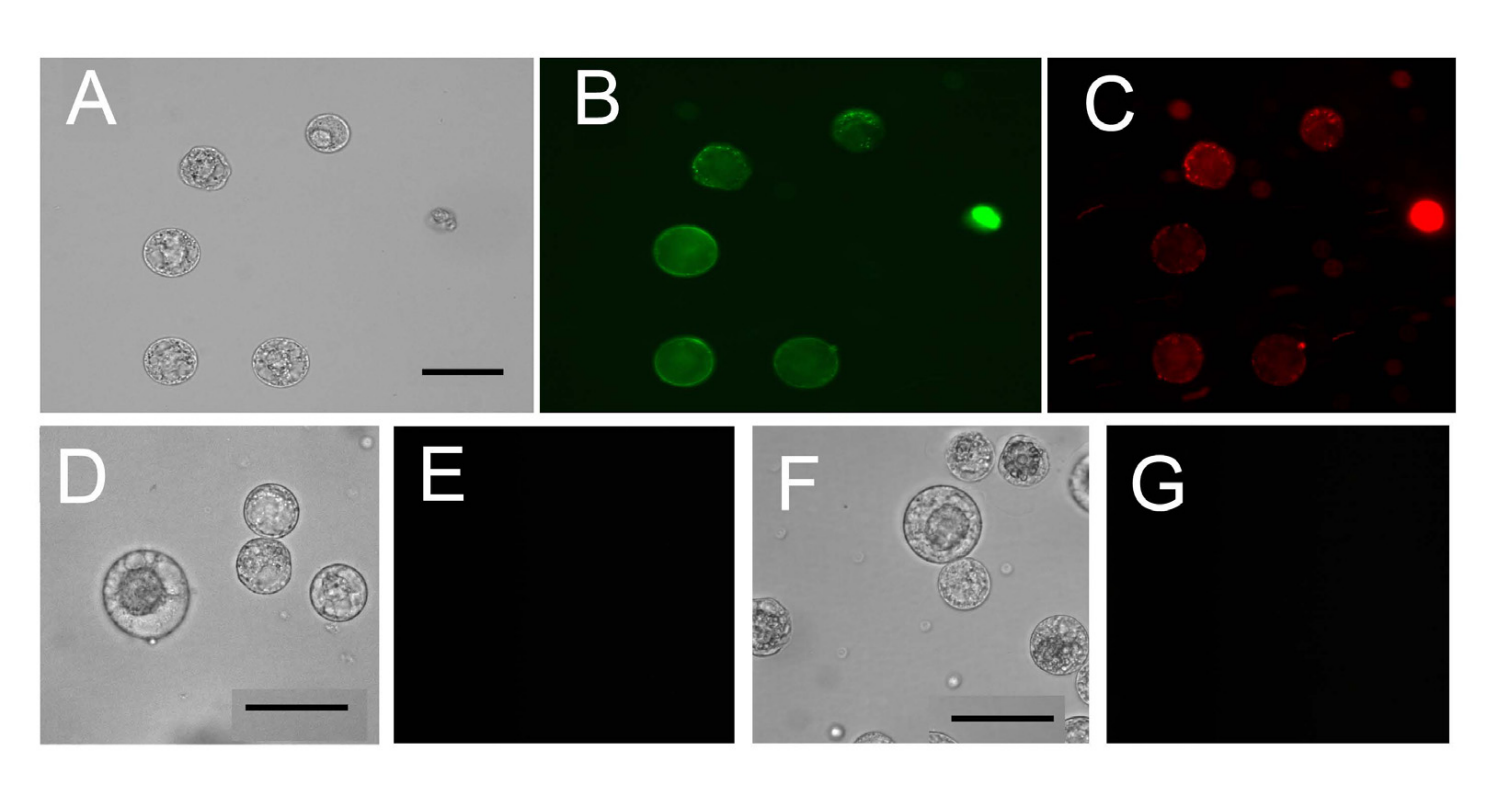
**CPNs deliver siGLO Red small RNAs to protoplasts**. Bright field and epifluorescence images of protoplasts treated with: **(A, B, C) **25 μM CPNs and 200 nM siGLO Red small RNAs (red); **(D, E) **only 200 nM siGLO Red small RNAs; **(F, G) **untreated protoplasts (negative controls). **(E) **Image shows no uptake of small RNAs in the absence of CPNs. **(G) **Image shows no green fluorescence, as expected. Experiments were repeated 2-3 times. Scale bars equal 20 μm.

**Figure 5:**
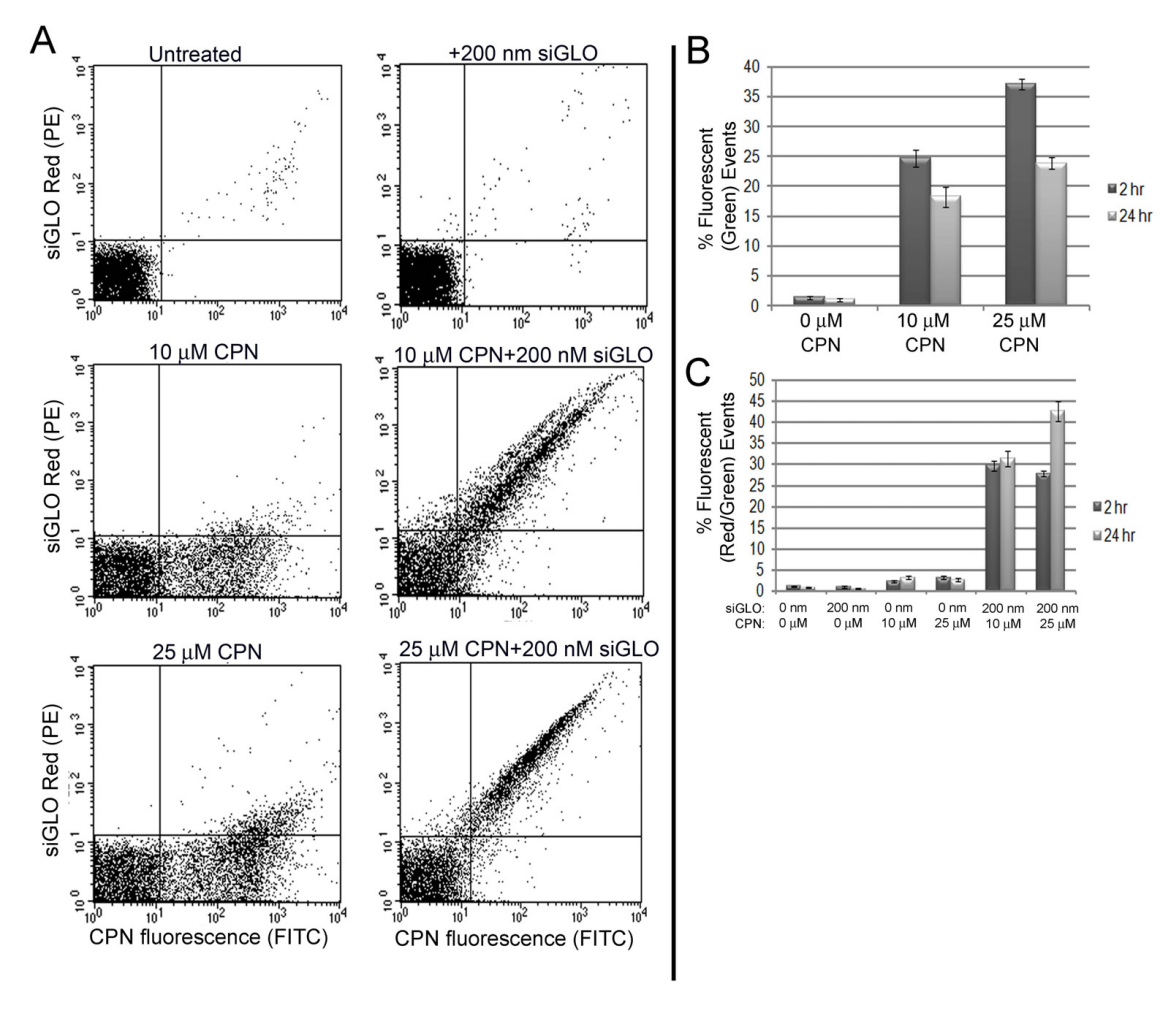
**Presence of CPN and siGLO Red small RNAs in protoplasts**. **(A) **Dot plots of BY-2 protoplasts cultured with medium only, 200 nM siGLO Red, 10 μM CPNs, 10 μM CPNs + 200 nM siGLO Red, 25 μM CPNs, or 25 μM CPNs+200 nM siGLO Red. CPN fluorescence is detected with FITC filter (x axis) and siGLO Red fluorescence (y axis) is detected with PE filter in protoplasts cultured for 24 h. Each dot represents a single event with emissions frequency that is the combination of the fluorophores. The gated population in the lower left quadrant represents the majority of nonfluorescent cells. The upper right quadrant represents the majority of events that contain both green and red fluorescence due to CPNs and siGLO Red small RNAs. The upper left quadrant represent events that are positive only for siGLO Red small RNAs and the lower right quadrant represent events that are positive for only CPNs. Highly fluorescent protoplasts are located furthest along the x- and y- axes. **(B) **Bar graph reports the average of 10 replicate experiments using FACs to record the number of green fluorescent events inside protoplasts, as an indication of the internalization of CPNs (lower right quadrant of dot plots). Samples were treated with 0, 10, or 25 μM CPNs and then incubated for 2 and 24 h. Between 18-37% of protoplasts produce positive events via cytometric analyses. **(C) **Bar graph reports the average and stand deviations of 10 replicate experiments, recording the number of events reporting internalization of both CPNs and siGLO Red (upper right quadrant of the dot plots). Between 27 and 42% of recorded events are positive for both CPNs and siGLO Red RNAs when they are co-delivered to protoplasts.

Commercially available siGLO Red are fluorescently labeled RNA duplexes which were combined with CPNs and delivered to BY-2 protoplast culture medium to assess protoplast transfection. Both green and red fluorescence, which corresponds to CPNs and siGLO Red, respectively, were seen inside BY-2 protoplasts within 2 h of delivery (Figure 4). FACS methods were employed to: a) measure the fluorescence intensity in each cell b) detect and count the number of fluorescentprotoplasts in large populations (10,000 protoplasts). A set of untreated BY-2 protoplasts without CPN treatment were gated to represent the major non-fluorescent population (Figure 5A). FACS demonstrated that 10 or 25 μM CPNs penetrated BY-2 protoplasts following treatment for 2 or 24 h. Clearly, not all protoplasts showed CPN-uptake. However comparing treatments at 2 and 24 h, there was undoubtedly an increase in CPN-uptake by protoplasts that was concentration and time dependent (Figure 5B).

Cytometric analysis also showed that siGLO Red failed to penetrate protoplasts in the absence of CPNs (Figure 5A). However, combining 10 μM or 25 μM CPNs with 200 nM siGLO Red, there is a significant and positive shift in the number of events containing red fluorescence (Figure 5A, C). The combined epifluorescence microscopic and cytometric analyses (Figures 4 and 5) indicate that CPNs were responsible for siGLO Red uptake.

The ability of CPNs to deliver siRNAs targeting an endogenous gene was also examined. siRNAs were generated to the plant cellulose synthase gene, *NtCesA-1*, and CPN-siRNA complexes were delivered to protoplasts. Under suitable media conditions, BY-2 protoplasts can regenerate their cell walls during 3 d of culture [30,31]. *NtCesA-1 *is a central factor in cell wall deposition in plants and therefore we hypothesized that knocking down *NtCesA-1 *expression would block cell wall regeneration in protoplasts. Given that the flow cytometry data shows ~ 40% uptake of siGLO Red accompanied by CPNs, it is possible that a similar population of protoplasts received *CesA-1 *siRNAs. We employed calcofluor white M2R staining [32] to assess cell wall regeneration following silencing *NtCesA-1a *and propidium iodide staining to monitor viability [33].

**Figure 6:**
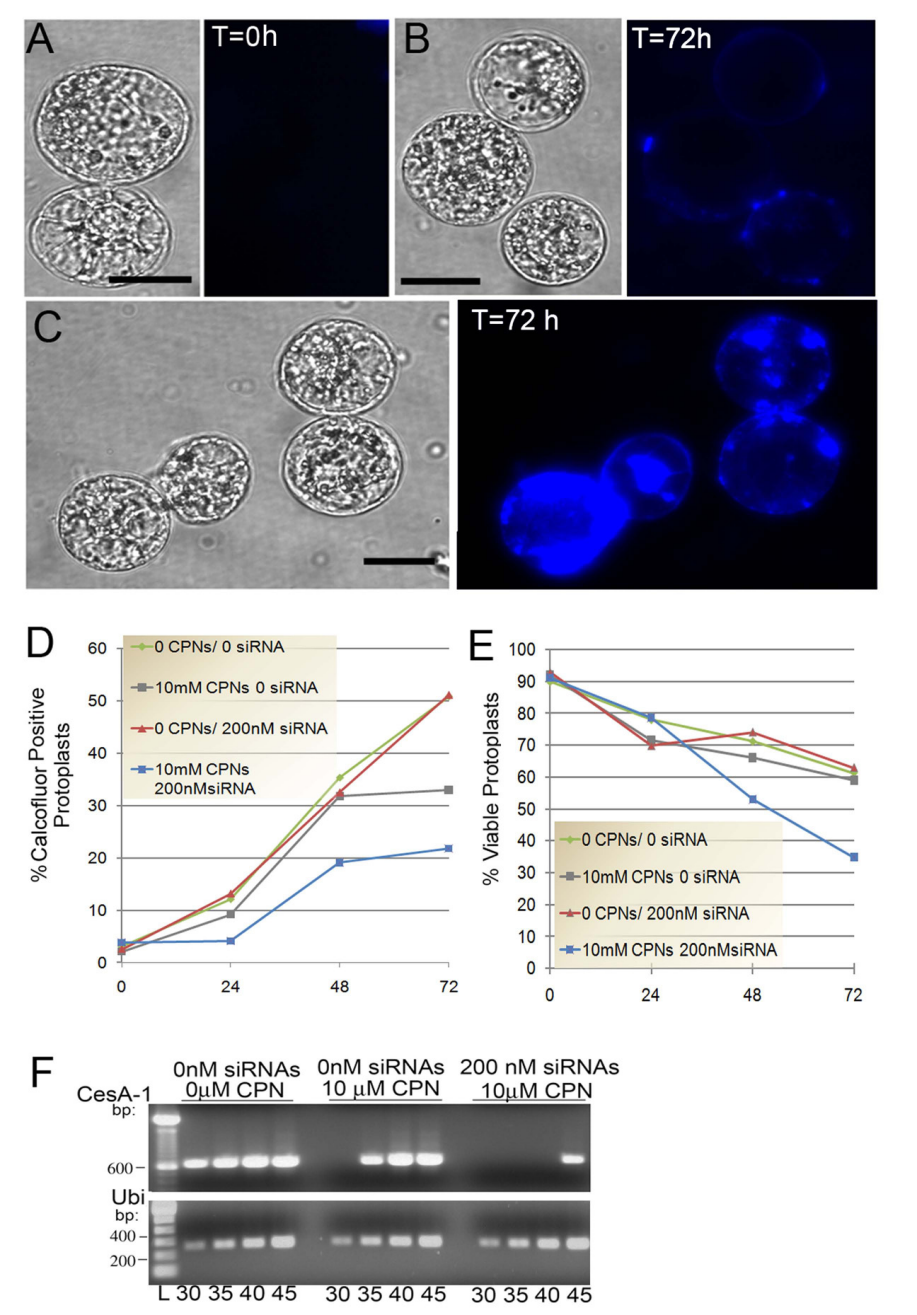
**CPN delivery of *CesA-1 *siRNAs suppress cell wall regeneration**. **(A) **Protoplasts were harvested and immediately stained with calcofluor white (T = 0 h) to verify complete digestion and elimination of cell walls. Image shows no calcofluor fluorescence. **(B) **Protoplasts at 72 h treated with 10 μM CPNs and 200 nM *NtCesA-1 *siRNAs show few faint patches of blue fluorescence at the plasma membrane. **(C) **Untreated protoplasts at 72 h show significant deposition of cellulose at the cell surface. Experiments were repeated five times. Scale bars represent 10 μm. **(D) **The average percent of protoplasts from two experiments that showed calcofluor staining at 0, 24, 48, and 72 h following treatment with CPNs and *NtCesA-1 *siRNAs. **(E) **Propidium iodide was used to determine the percent viable protoplasts at 0, 24, 48, and 72 h following treatment with CPNs and *NtCesA-1 *siRNAs. Averages were determined for three replicate experiments. **(F) **Ethidium bromide- stained 1% agarose gels containing semi quantitative RT-PCR products detecting *NtCesA-1 *or ubiquitin (Ubi) gene expression. The treatments with siRNAs and CPNs are indicated above each panel and the numbers of PCR cycles from 30-45 are indicated below each lane. Lane "L" indicates DNA ladder at the bottom of the gel and size (bp) markers are indicated on the left. As a control, semi-quantitative PCR shows ubiquitin gene expression.

Calcofluor white M2R staining conducted upon the completion of cell wall digestion (T = 0 h) confirms that there was no residual cell wall material remaining along protoplast surfaces (Figure 6A). At 72 h, greater amounts of calcofluor white M2R fluorescence was observed at the margins of untreated protoplasts indicating that the culture conditions were suitable for cell wall regeneration (Figure 6C). For siRNA and CPN treated protoplasts, calcofluor staining was significantly reduced to a level that was barely visible. Rare, minor patches of cellulose occurred along the plasma membrane of some protoplasts at 72 h (Figure 6B). Interestingly, we tested CPN-siRNA complexes formed by mixing for 3 h and overnight at 4°C and we noted that the outcome of calcofluor staining was significantly reduced using CPN-siRNA complexes that were prepared by overnight incubation (data not shown). It is worth speculating that the overnight incubation led to maximal incorporation of siRNAs into complexes.

To determine the effectiveness of *NtCesA-1 *siRNA- delivery in blocking cell wall regeneration, we recorded the average percentage of calcofluor positive protoplasts relative to the total number of living protoplasts counted (n = 400 protoplasts) in two replicate experiments. Protoplast viability was confirmed using propidium iodide (see below). For protoplasts that were untreated (no siRNA or CPN) or treated only with *NtCesA-1 *siRNAs, 51-54% were calcofluor positively by 72 h. Treating protoplasts with CPN or siRNA alone had no effect on cell wall regeneration until the time period between 48-72 h. There was a steady increase in the proportion of untreated or siRNA-treated protoplasts that stained positive over time (Figure 6D). For protoplasts treated with CPN alone, there is a slight plateau between 48 and 72 h (19-23%) and only a few faint patches of newly synthesized cell walls were seen in those protoplasts (Figure 6B and 6D) [33]. However, this is contrasted by protoplasts treated with 10 μM CPNs and 200 nM *NtCesA-1 *siRNAs which showed no change in cell wall regeneration between 0 and 24 h followed by a slow increase in calcofluor staining until 48 h. There appeared to be a plateau between 48 and 72 h where cell wall regeneration did not continue in a manner similar to untreated protoplasts (Figure 6C). Calcofluor staining was seen in 33-38% of protoplasts at 48 and 72 h, suggesting that CPN treatment could hamper growth at later times.

Propidium iodide was used to determine the percent viable protoplasts harvested at 0, 24, 48, and 72 h (Figure 6E). Propidium iodide stains nonviable protoplasts and cells, shows absorption/emission maximum at 536/617 nm, and was employed to measure the impact of CPNs on cell viability. We counted populations of 100-250 protoplasts to determine the proportions that were propidium iodide positive and/or contained CPNs. Untreated protoplasts show a slow decline in viability from 90% at 0 h to ~ 60% at 72 h. Protoplasts that were treated with only CPNs or only siRNAs showed comparable levels of decline. However, protoplasts treated with both CPNs and siRNAs showed a significant drop in viability between 24 and 72 h with ~ 35% of protoplasts remaining alive (Figure 6E). These data suggest that cellulose synthase activity is essential for extending the lifetime of protoplast. The effect of CPN plus siRNAs on cell viability and deposition of cellulose on the cell surface [33] indicates that CPNs were effective vehicle for siRNA delivery and targeted downregulation of *NtCesA-1*expression.

Knockdown of *NtCesA-1a *transcript accumulation was confirmed by semi-quantitative PCR (Figure 6F). *NtCesA-1a *silenced protoplasts were harvested at 48 h post delivery of CPNs alone and 200 nM siRNAs plus CPNs. The messages were reduced >17% and >76% compared with untreated control samples. Ubiquitin mRNA served as an internal control for RNA quality and RT-PCR amplification, and the mRNA levels were similar (Figure 6F).

## Discussion

CPNs are fluorescent conjugated polymer nanoparticles and are valuable for live plant cell imaging. Their inherent photophysical properties include high fluorescence quantum yield, large extinction coefficient, and efficient optical signal transduction making them a superior choice for biological imaging [22,23]. Furthermore, we demonstrate that CPNs are an effective transfection vehicle for delivery of siRNAs into plant protoplast. Other transfection methods that are widely employed for delivery of nucleic acids to plant protoplasts include electroporation, polyethylene glycol, and lipofectamine [34-36], and only electroporation and polyethylene glycol has been used for direct delivery of siRNAs [37,38]. With respect to electroporation, the electric pulse can cause an immediate loss of up to 40% viable protoplast [35,39]. This much greater loss than following the use of CPN-delivery, which causes only 5-20% loss of viability within the first 24 h of delivery (Table 1). Unlike the CPN delivery method, the optimal conditions for delivery of siRNAs by electroporation require extensive optimization [34] of the voltage and pulse time to ensure high transfection rates. Therefore CPNs are attractive and facile choice for efficient siRNA delivery into protoplasts without compromising cell viability. Furthermore, the intrinsic fluorescence enables real time detection of CPN-uptake by protoplasts and offers the opportunity to monitor the rate of cellular responses following siRNA uptake in synchronously treated cells.

Flow cytometry for studying physiological events or dye uptake in plant protoplasts has been used in recent years. Protoplast morphology and the distribution of light-scattering intensities can vary widely for different species and cell cultures and this can impact the quality of the results. We examined green and red fluorescence (FL1-H and FL4-H) and expected to detect a minor population of dots that would result from cell debris or dying protoplasts. Notably the FACS results (Figure 5B) and manual counting of CPN-uptake by protoplasts produced somewhat different quantitative outcomes. A maximum of 35% of protoplasts were green fluorescent following treatment with 25 μM CPNs for 2 hrs as measure by FACS, but under the microscope we noted 60-75% of protoplasts were green fluorescent. One explanation is that the larger population that was sorted by FACS led to a broader and unbiased assessment. Another possibility is that the CPN fluorescence inside some cells might be low and might overlap with the autofluorescence of the gated population. Thus fewer CPN-positive protoplasts may be detected by FACS than by manual counting. A third explanation for the low counts by FACS is that the method of mixing protoplasts with CPNs may require further optimization to ensure a broader population is exposed to CPNs. Perhaps placing the culture on a rotary shaker at low speed would enhance mixing of protoplasts and CPNs and could increase the percentage of transfected protoplasts. We also observed CPNs binding to debris in the cell cultures. The nature of the debris is a mixture of lysed cell constituents and cell wall materials. We know from examining CPN-treated BY-2 cells that CPNs bind easily to plant cell walls. Therefore, the presence of cell wall material in the cultures likely depletes CPNs that could transfect protoplasts. Perhaps improved handling of protoplast preparations through further filtration or washing could reduce cell wall debris and enhance the percentage of transfected protoplasts. Further experiments are needed to determine the conditions for improved CPN internalization.

Cell wall biosynthesis is central to cell elongation, plant growth and development and new methodologies to modify the cellulose and lignin content could be employed for generating genetically improved plants [40]. Researchers are working on agronomically important crops to increase cellulose content and decrease lignin content to improve forage digestibility and improve the use of crops as biofuels for ethanol production. The biosynthetic pathways leading to cell wall deposition (cellulose and lignin) and assembly into a functional wall are not well described [40]. Significant advances have been made in recent years with the identification of *CesA *genes that encode the catalytic subunits for cellulose synthase. In *Arabidopsis thaliana*, there are 10 members of the *AtCESA *gene family and only three genes *AtCESA1, AtCESA3 *and *AtCESA6 *are known to be important for primary cell wall synthesis [41]. *CesA *subunits assemble in rosettes and these rosettes produce glucan chains. The rosettes assemble into microfibrils along the plasma membrane [40]. Patches of cellulose occurring along the plasma membrane can be seen by calcofluor staining [33]. In our experiments, we noted that at 72 h in untreated BY-2 protoplasts, there were patches of cellulose which coalesced to form larger areas of deposited primary cell wall (Figure 6C). Chemicals such as ancymidol and isoxaben are known to inhibit cell wall production and have been employed to increase our knowledge of the mechanisms controlling cellulose biosynthesis and deposition along the plasma membrane. One drawback of using a chemical approach to knockdown cellulose synthase is that they can have additional impacts on other subcellular functions [42]. For example, isoxaben disrupts microtubule organization as well as inhibits the synthesis of cellulose microfibrils [43]. Therefore, siRNA delivery targeting specific genes in the cellulose biosynthesis pathway is preferred to examine the role of each gene in cell wall deposition. Recently, siRNA delivery via viral vectors (*Potato virus X, Barley stripe mosaic virus*) to intact *N. benthamiana *and barley plants has provided valuable evidence that RNAi technology can be employed to knockdown *CesA-1*, *CesA-2*, and *CesA-6 *gene expression [25,44]. By silencing individual members of the CesA gene family researchers were able to determine which genes are primary contributors to cell wall formation. In addition, plants showed compensatory changes in polysaccharide composition of the cell walls and this demonstrated that an RNAi approach created further opportunities to explore the relationships between the cellulose synthase genes and pectin biosynthesis [25,44]. Importantly, using viral vectors to deliver siRNAs into protoplasts may not be advisable because the viruses themselves may impact protoplast viability and cell wall regeneration. In addition, viral delivery relies on electroporation or PEG transfection methods which may also impact protoplast viability. In this study, we employed a CPN-siRNA delivery method that is taken up by protoplasts in a manner that does not hamper viability and had no obvious effect on cell wall regeneration during the first 48 h. CPN delivery of *CesA-1 *siRNAs was effective for silencing cellulose synthase in protoplasts showing that this technology can be used to monitor the early stages of cellulose deposition. When we compare Figure 6D and 6E, we note that there is reduced protoplast viability along with hampered cellulose synthesis. Protoplasts left untreated, treated with siRNAs only (no CPNs), or CPNs only (no siRNAs) showed comparable levels of cell wall regeneration and viability. Only the addition of 10 μM CPNs plus 200 nM siRNAs caused a significant loss in viability and cell wall regeneration. Calcofluor staining showed no obvious buildup of cellulosic patches along the plasma membrane, while untreated samples showed greater regeneration. We also realize that there is significant sequence similarity among tobacco *CesA *genes and that the long piece of *CesA *used to generate the siRNAs might directly knock down multiple *CesA *genes. The correlation suggests that silencing *CesA *genes reduced protoplast viability, indicating that cellulose synthesis is an important housekeeping function. Events that slow regeneration or hamper cell expansion could lead to loss of viability. Complete and speedy regeneration of the primary cell wall might be important for protoplasts to respond to the osmotic pressure of the medium and remain alive. Future research is needed to understand the link between *CesA *gene function and cell viability. These outcomes show that CPN delivery methods may be valuable for such studies in protoplasts.

Animal cells take up extracellular materials by at least four different routes: receptor-mediated endocytosis, non-ionic diffusion, carrier mediated uptake, and facilitated diffusion. These routes are largely unexplored in plants, and little is known about the compartments carrying endocytic cargo from the plant plasma membrane [45]. Several approaches have been used to identify specific compartments in the endomembrane system. First is the use of immune electron microscopy and antibodies recognizing known markers to identify the compartment. Second is the use of organelle reporters such as GFP fused to organellar targeted protein domains [46-48]. Third is the use of FM4-64 fluorescent styryl dyes which are taken up by endocytic vesicles and are used to follow the vesicle trafficking network to Golgi and prevacuolar vesicles [49,50]. Such tools have led researchers to determine that the pathway from the early endosome to prevacuolar/multivesiculate bodies in animals and plants differ. In animals, the multivesiculate bodies send cargo to the lysosome while in plants they deliver their cargo to the vacuole. The plant prevacuolar vesicles can deliver cargo to storage vacuoles as well as the lytic compartment [51]. The recycling endosome is a compartment that returns cargo to the plasma membrane and is well described in animal cells but is relatively unknown in plant cells, although FM4-64 staining suggests plants likely have these types of vesicles [50,27]. Recently, nanogold particles have been used to probe the plant endocytic pathways [26]. Detailed electron microscopic analysis of BY-2 protoplasts treated with positively and negatively charged nanogold particles revealed the presence of clathrin-dependent and -independent pathways that lead to degradation or recycling [26]. In Figure 2 we report the pattern of CPN localization in granules near the cell surface followed by accumulation in larger vesicles, and this coincides with the pattern of FM4-64 staining. The FM4-64 staining was more pronounced in CPN-treated cells than in nontreated cells suggesting that dye uptake was stimulated by CPNs. Further research using confocal microscopy will be needed to follow the true path of CPN internalization, and to learn if this pathway involves receptor mediated endocytosis or an alternative carrier mediated uptake that coincidently leads to increased internal staining by FM4-64. In addition, the possibility of CPNs entering the prevacuolar compartment is intriguing, given that we show in later experiments CPN-siRNA complexes are effective for PTGS. Based on these data, it is worth speculating that if CPNs-siRNAs complexes are endocytosed, that they are then released into the cytoplasm. Perhaps there are cellular conditions that enable siRNAs to dissociate from CPNs and to become active in the silencing pathway. Therefore the route for CPNs to enter prevacuolar vesicles from the endosome or cytoplasm is not made obvious by these experiments. If CPN uptake is via the endosome, then one explanation is that siRNAs exit the endosome while CPNs remain and are transported to the prevacuolar compartment. Perhaps a shift in the pH of the endocytic compartment causes dissociation of the complex. An alternative route is that CPN-siRNA complexes exit the endosome, dissociate in the cytoplasm, and then CPNs enter the prevacuolar compartment by an undefined mechanism.

In total, CPNs present a promising tool for live cell imaging of the endocytic trafficking pathways in plants. This study shows that we can deliver positively charged CPNs to protoplast culture medium and because of their intrinsic fluorescence we can visually monitor the route of uptake. Future research will examine the possibility of recording the trafficking of CPNs from the plasma membrane to and within the endocytic pathway alongside fluorophores fused to endocytic markers. This is an advance over the use of fusion proteins which are often transgenically expressed. One limitation to employing transgenic plants is that the protein fusions are synthesized within the cell and their pathway to the plasma membrane potentially overlaps with their pathway of recycling from the plasma membrane back into the cell. On the other hand CPNs can be delivered into the culture medium and their intrinsic fluorescence can be relied on to visually monitor the trafficking of molecules within the endocytic pathway to obtain new information about these pathways.

## Conclusions

CPNs represent a significant advance in technology for the delivery of siRNAs to plant protoplasts. Other methods of siRNA delivery include the use of viral vectors, electroporation, and polyethylene glycol. For example, one advantage of CPNs-siRNA complexes over the use of viral vectors is the ability to deliver siRNAs that knockdown expression of genes that are vital cellular functions without concern for viral pathology affecting experimental outcomes. With respect to electroporation and polyethylene glycol, it is possible that CPNs may have a lower impact on protoplasts viability, although further experiments are needed to compare CPN-mediated transfer of siRNAs with these other methods to determine which are less disruptive to cell functions. Because CPNs are nontoxic to protoplasts and are easily added to culture medium, this technology could be adapted to high throughput applications. It would be straightforward to synthesize siRNAs targeting various regulatory steps in a pathway, deliver CPN-siRNA complexes to protoplasts, and then monitor the outcomes of suppressing housekeeping genes on cellular functions.

We also learned that CPNs could be a valuable imaging tool for plant biology. The endocytic pathway is not as well explored in plants as in vertebrate systems. Live cell imaging recording trafficking of CPNs via the endocytic pathway could yield valuable new information about membrane transport in plants.

## Methods

### Synthesis of CPNs

**Figure 7:**
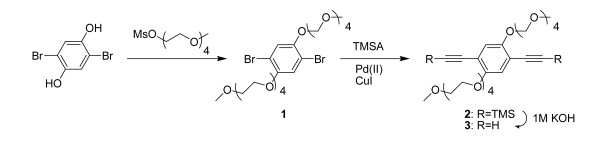
**Fabrication of compounds 1, 2, and 3**.

The fabrication of CPNs was previously described [23] (Figure 7). Briefly, an amine-containing poly(phenylene ethynylene) (PPE) was synthesized by polymerizing 13,13'-(2,5-diethynyl-1,4-phenylene)bis(oxy)bis(2,5,8,11-tetraoxatridecane) and 2,2'(2,2'-(2,5-dibromo-1,4-phenylene)bis(ethane-2,1-diyl))bis(oxy)diethanamine in a mixed solvent of N-methyl pyrrolidone and morpholine using palladium/copper catalysts. The PPE solution was treated with excess amounts of glacial acetic acid followed by dialysis (10,000 MWCO) against dH_2_O. Final PPE- dH_2_O solution was filtered using a syringe filter (0.45 μm) and stored at room temperature.

Compound (1) is 13,13'-(2,5-dibromo-1,4-phenylene)bis(oxy)bis(2,5,8,11-tetraoxatridecane) and was prepared by incubating at 80°C overnight a suspension of 2-{2-[2-(2-methoxyethoxy)ethoxy]ethoxy}thyl 4-methylbenzenesulfonate^1 ^(20.6 mM; 5.9 g), 2,5-dibromohydroquinone (10.3 mMl; 2.76 g) and K_2_CO_3 _(103 mM; 14 g) in 30 mL of dimethyl formamide (DMF). The mixture was concentrated in vacuo and diluted with 50 mL of dichloromethane. The solution was washed with three times with 20 mL dH_2_O, dried over Na_2_SO_4_, and evaporated in vacuo. The crude product was purified by column chromatography (silica gel, ethyl acetate/hexane (3:1, v/v). Yield : 3.7 g (55%). ^1^H NMR(600 MHz) : δ = 7.15 (s, 1H, Ar-H, *J *= 6), 4.12 (t, 4H, Ar-OCH_2_, *J *= 6), 3.87 (t, 4H, OCH_2_, *J *= 6), 3.76 (t, 4H, OCH_2_, *J *= 6), 3.69-3.63 (m, 16H, OCH_2_), 3.55 (t, 4H, OCH_2_, *J *= 6), 3.37 (s, 6H, CH_3_); 13C NMR(150 MHz) : δ = 150.5, 119.4, 111.6, 72.1, 71.3, 70.9, 70.8, 70.72, 70.4, 69.8, 59.2.

Compound (2) is (2,5-bis(2,5,8,11-tetraoxatridecan-13-yloxy)-1,4-phenylene)bis(ethyne-2,1-diyl)bis(trimethylsilane) and was prepared by adding Compound (1) (5 g, 7.7 mM) to a shrink flask fitted with a stir bar and Pd(PPh_3_)_2_Cl_2 _(0.54 g, 0.77 mM) and CuI (0.073 g, 0.39 mM). Thirty mL of a 2:1 mixture of tetrahydrofuran and diisopropylamine was added to the reaction. Following addition of trimethylsilylacetylene (4.4 mL, 31 mM), the reaction mixture was heated to 60°C for 12 h. After evaporating solvent, the crude mixture was dissolved in methylenechloride and washed twice with 30 mL saturated ammonium chloride, followed by drying over anhydrous MgSO_4_. The solvent was evaporated to produce dark brown oil, which was purified by column chromatography (silica gel, ethyl acetate/hexane (4:1, v/v)). Yield : 5 g (95%). ^1^H NMR(600 MHz) : δ = 6.91 (s, 1H, Ar-H), 4.12 (t, 4H, Ar-OCH_2_, *J *= 6), 3.87 (t, 4H, OCH_2_, *J *= 6), 3.78 (t, 4H, OCH_2_, *J *= 6), 3.68-3.66 (m, 12H, OCH_2_), 3.64 (t, 4H, OCH_2_, *J *= 6), 3.54 (t, 4H, OCH_2_, *J *= 6), 3.38 (s, 6H, CH_3_), 0.25 (s, 9H, SiMe_3_); 13C NMR(150 MHz) : δ = 154.0, 117.9, 114.3, 100.9, 100.4, 72.0, 71.2, 70.8, 70.7, 70.6, 69.7, 69.6, 59.0, 0.0.

Compound (3) is 13,13'-(2,5-diethynyl-1,4-phenylene)bis(oxy)bis(2,5,8,11-tetraoxatridecane). A 100 mL round-flask was charged with the compound (2) (2.5 g, 3.7 mM), 50 mL tetrahydrofuran, and 10 mL MeOH. Then 5 mL of 1 M KOH (*aq*) solution was added to the reaction mixture and stirred for 2 h. The solvent was evaporated and the reaction mixture was purified by column chromatography (silica gel, ethyl acetate). Yield : 1.1 g (90%). ^1^H NMR(400 MHz) : δ = 7.00 (s, 1H, Ar-H), 4.15 (t, 4H, Ar-OCH_2_, *J *= 6), 3.87 (t, 4H, OCH_2_, *J *= 6), 3.76 (dd, 4H, OCH_2_, *J *= 6), 3.73-3.63 (m, 16H, OCH_2_, *J *= 6), 3.55 (dd, 4H, OCH_2_, *J *= 6), 3.38 (s, 6H, CH_3_, *J *= 6), 3.35 (s, 2H, CH); 13C NMR(150 MHz) : δ = 154.1, 118.3, 113.6, 82.8, 79.6, 71.9, 71.1, 70.7, 70.6, 70.5, 69.6, 69.5,59.0.

### **Preparation of siRNAs for cellulose synthases**, ***NtCesA-1a *and *NtCesA-1b***

TRIzol^® ^reagent was used to extract total RNA from tobacco (*N. tabacum*) leaves (Invitrogen Corp, Carlsbad, CA). cDNA of the putative cellulose synthase (AF233892), *NtCesA-1a *and *NtCesA-1b *was prepared using Superscript III reverse transcriptase (Life Technologies) and then a fragment of the gene was PCR amplified (640 base pairs (bp) using forward (5'- AGTGTATGTGGGTACCGGATG- 3') and reverse (5'- CCATATGGGACA ATGCCTAC - 3') primers. Following a 5-min denaturation at 94°C, PCR was performed for 34 cycles of 94°C for 2 min, 94°C for 15 s, 50°C for 30 s, and 68°C for 45 s, followed by final 5-min extension at 68°C [25]. The 640 bp PCR product was purified using PCR Preps DNA Purification system (Promega, Madison,WI) and cloned into the pGEM-T Easy vector (Promega) according to manufacturer's instructions. The nucleotide sequence of this cDNA fragment was confirmed as 100% and 98% identical for *NtCesA-1a *and *NtCesA-1b*, respectively. To generate sense and anti sense RNA, pGEM-T:*NtCesA *was linearized using *Nco*I or *Sal*I and *in vitro *transcription was performed (RiboMAX, Promega) with SP6 and T7 RNA polymerases. Transcription products were purified using MEGAclear Kit (Ambion, Austin, TX). Sense and anti sense RNAs were annealed in annealing buffer (100 mM,KOAc, 4 mM MgCl_2 _and 60 mM HEPES- KOH, pH 7.4), boiled for 5 min, and incubated overnight at 37°C [37]. The resulting double strand RNAs were precipitated using ethanol and then dissolved in nuclease-free double distilled (dd) H_2_O. siRNAs were generated by treating double stranded *NtCesA*-1 with recombinant Dicer enzyme according to the manufacturer's instructions (Gene Therapy Systems, San Diego, CA). The reaction was stopped by adding the Dicer stop solution and 22 bp products were detected using 3% agarose gel electrophoresis [37]. The final siRNA products were purified using RNA purification column 1(Gene Therapy Systems) and dissolved in nuclease-free ddH_2_O.

### BY-2 protoplast preparation

Typically, BY-2 cells are grown are subcultured from a 4 d-old liquid culture by transferring 10 mL BY-2 cells to 40 mL fresh BY-2 culture medium (Murashige and Skoog salts pH 5.6 (Sigma-Aldrich Co, St. Louis, MO), 30 g.L^-1 ^sucrose, 256 mg.L^-1 ^KH_2_PO_4_, 100 mg.L^-1 ^myo-inositol, 1 mg.L^-1 ^thiamine, and 0.2 mg.L^-1 ^2,4-dichlorophenoxyacetic acid). Cells are grown on a rotary shaker that is maintained in the dark at 120 rpm at 28°C.

Protoplasts were prepared from 3 d old tobacco BY-2 suspension cells using standard methods [52,53]. Ten ml of packed BY-2 cells (sedimented by centrifugation at 100-g for 5 min) were resuspended in 100 mL of enzyme solution (1.5% cellulase "Onozuka RS" (Yakult Pharmaceutical Ind. Co. Ltd., Tokyo, Japan), 0.2% macerase (Calbiochem-Novabiochem Corp., La Jolla, CA), 0.5 M mannitol, and 3.6 mM 2-(N-morpholino) ethanesulfonic acid (pH 5.5) ) in a 1 L flask and incubated for 3-4 h at 28°C on a rotary shaker at 100 rpm. Protoplasts were recovered by filtration through 41 μm nylon mesh (Spectrum Laboratories, Inc., Rancho Dominguez, CA), and washed twice with Protoplast Wash Solution (0.5 M mannitol, 3.6 mM 2-(N-morpholino) ethanesulfonic acid (pH 5.5) at 59 g for 5 min. Protoplasts were resuspended in Protoplast Resuspension Solution (BY-2 culture media plus 0.45 M mannitol) to a density about 1 × 10^6 ^protoplasts mL^-1^. Protoplast viability was measured using 0.1% fluorescein diacetate prepared in 1 ml of 50 mM phosphate buffer (pH 7.4). For siRNA delivery experiments, protoplasts were cultured in cell wall regeneration medium.

### siRNA delivery to protoplasts and intact BY-2 cells

Protoplasts (1 × 10^6^mL^-1^) or intact BY-2 cells (1 × 10^6^mL^-1^) were mixed with CPNs in Protoplast Resuspension Solution to a final concentration of 0, 5, 10, 15 and 20 μM and added to 6-well culture plates (Corning Inc., Corning, NY). Each well of the culture plate was lined with Protoplast Resuspension Solution plus 1.0% agarose (pH 5.6). Protoplasts and BY-2 cells were cultured at 28°C. Samples were harvested at 2, 5, 10 and 24 h and the proportion of CPN containing protoplasts and intact BY-2 cells were determined using a haemocytometer. In addition, the proportion of protoplasts or cells for which fluorescence was seen to be associating with the cell wall, plasma membrane, nucleus, and/or cytoplasm was recorded.

Protoplasts were resuspended in Protoplast Resuspension Solution (BY-2 culture media plus 0.45 M mannitol) to a density about 1 × 10^5 ^protoplasts.mL^-1^. CPNs (10 or 25 μM) were incubated with 200 nM siGLO Red for 3 h (Thermo Fisher Scientific, Pittsburgh, PA) and the complex was delivered to protoplasts. CPNs were incubated with 200 nM *NtCesA-1 *siRNAs overnight and then delivered to protoplasts. We found that mixing CPNs and siGLO Red for 3 h was sufficient to demonstrate transfection of CPN-siRNA complexes, but overnight mixing of CPNs and *NtCesA-1 *siRNAs improved complexation and improved siRNA delivery to plant protoplasts resulting in a measurable phenotype. Protoplast cultures were maintained in the dark at 28°C for 2 h and 24 h. Controls included treating protoplasts with Protoplast Resuspension Solution, 200 nM siGLO Red only, *NtCesA-1 *siRNAs or CPNs only. The protoplast containing both CPNs and siGLO Red were counted using a haemocytometer.

### Propidium iodide and FM4-64 dye treatment

Propidium iodide, contained in the Plant Cell Viability Assay Kit (Sigma-Aldrich Co), was solubilized according to manufacturer's instructions. FM4-64 (Invitrogen Corp) staining was carried out to monitor CPN uptake, as previously described [54]. Protoplasts (1 × 10^5^.mL^-1^) were incubated with 10 μM CPNs for 24 h at 28°C. 20,000 protoplasts (which were previously treated with medium or 10 μM CPNs and incubated for 24 h at 28°C) were incubated with 10 μM FM4-64 at room temperature for 10 min and then monitored using confocal microscopy.

### Epifluorescence and confocal microscopy

A Nikon E600 (Nikon Corp., Tokyo, Japan) epifluorescence microscope with a B2A filter cube (470- to 490-nm excitation filter), a DM505 dichroic mirror, and a BA520 barrier filter was used to monitor FDA staining following enzymatic digestion of BY-2 cells and to study uptake of CPNs protoplasts and intact BY-2 cells. Propidium iodide was detected in protoplasts using a UV filter cube. siGLO Red fluorescence (absorption/emission maximum at 557 nm/570 nm) was viewed using a Y-2E/C TX red filter cube containing a 540- to 580-nm excitation filter, a DM595 dichroic mirror, and a BA600-660 barrier filter. Images were captured using the Optronics Magnafire camera (Optronics Inc., Goleta, CA) and were edited using Adobe Photoshop software (Adobe Systems Inc., San Jose, CA). Haemocytometer observations were recorded using Microsoft Excel software.

A Leica TCS SP2 (Leica Microsystems, Bannockburn, IL) confocal imaging system attached to a Leica DME 14 upright microscope equipped with Ar/Kr lasers were used to study BY-2 cells treated with CPNs and FM4-64 staining protoplasts. Serial images were collected using 0.3 μm steps and 3-D images of 100 μm thick sections were compiled.

### Fluorescence activated cell sorting (FACS) flow-cytometry of treated BY-2 protoplasts

A Becton Dickinson FACS Calibur flow cytometer (Becton Dickinson, Franklin Lakes, NJ) equipped with an Ar laser (excitation of 488 nm) was used to assess CPN-uptake by protoplasts. Protoplasts (1 × 10^6^.mL^-1^) were mixed with 10 μM and 20 μM CPNs in Protoplast Resuspension Solution and added to 6-well culture plates (Corning Inc., Corning, NY) containing Protoplast Resuspension Solution plus 1.0% agarose (pH 5.7). Protoplasts treated with buffer, or a 1:1 mixture of untreated plus CPN-treated protoplasts were used as controls. Protoplasts were cultured at 28°C and FACS was performed at 2 h and 24 h of incubation.

The sorting capability of 10000 cells.s^-1 ^and fluorescence emission (FL1-H, FL2-H) was detected using a 530 nm and 585 nm band pass filters. The percentages of fluorescence-emitting protoplasts were assessed as evidence of CPNs and siGLO Red uptake by protoplasts. Data were analyzed on a Macintosh computer equipped with BD CellQuest Pro program (Becton Dickinson) and were presented as two dimensional dot plots which represent CPN fluorescence emissions on the X-axis and siGLO Red fluorescence emissions on Y- axis. Data was compiled using Adobe Photoshop software.

### Semi-quantitative RT-PCR of silenced protoplasts

Semi-quantitative RT-PCR was utilized to monitor *NtCesA *transcriptional levels following siRNA delivery. Extraction of total RNA from BY-2 protoplasts was carried out using SV Total RNA Isolation System (Promega, Madison, WI). The first strand cDNA was synthesized using SuperScript III reverse transcriptase (Invitrogen Corp), 1 μg total RNA and oligo(dT) primers. PCR was performed using *NtCesA*-1 specific forward primer (5'-AGTGTA TGTGGGTACCGGATG-3') and *NtCesA *reverse (5'-CCATATGGGACAATGCCTAC-3') primer that also shares homology with *NtCesA-2*. Forward (5'-GCCTCCGTGGTGGTG CTAAG- 3'), and reverse (5'-TCAATCGGCACC GGCCTT G-3') primers were used to amplify ubiquitin (AY912494) cDNA (261 bp) as the internal control. Following 10-min denaturation at 95°C, PCR was performed for 30, 35, 40, 45 cycles of 95°C for 15 s and 60°C for 60 s. PCR products were analyzed using ethidium bromide stained 1% agarose gel. Gels were scanned using Alpha Image system (Alpha Innotech, San Leandro, CA) and the images were recorded. Densitometry was performed by Alpha Ease FC software (Alpha Innotech).

## Abbreviations

CPN: conjugated polymer nanoparticles; PTGS: post-transcriptional gene silencing; ddH_2_O: double distilled water; miRNA: microRNA; siRNA: small interfering RNA; TRV: *Tobacco rattle virus*; T-DNA: *Agrobacterium tumefaciens *based plasmid that can transform plant tissues; dsRNA: double stranded RNA; CbLCV: *Cabbage leaf curl virus*; FDA: Fluorescein diacetate

## Authors' contributions

AS carried out plant protoplast experiments. JV and JM oversaw the analysis, design and implementation. AN carried out CPN preparations. JV and JM drafted the manuscript. All authors read and approved the final manuscript.
